# Akermanite bioceramics promote osteogenesis, angiogenesis and suppress osteoclastogenesis for osteoporotic bone regeneration

**DOI:** 10.1038/srep22005

**Published:** 2016-02-25

**Authors:** Lunguo Xia, Zhilan Yin, Lixia Mao, Xiuhui Wang, Jiaqiang Liu, Xinquan Jiang, Zhiyuan Zhang, Kaili Lin, Jiang Chang, Bing Fang

**Affiliations:** 1 Center of Craniofacial Orthodontics, Department of Oral and Cranio-maxillofacial Science, Ninth People’s Hospital Affiliated to Shanghai Jiao Tong University, School of Medicine, Shanghai 200011, China; 2 State Key Laboratory of High Performance Ceramics and Superfine Microstructure, Shanghai Institute of Ceramics, Chinese Academy of Sciences, Shanghai 200050, China; 3 Oral Bioengineering and regenerative medicine Lab, Shanghai Research Institute of Stomatology, Ninth People’s Hospital Affiliated to Shanghai Jiao Tong University, School of Medicine, Shanghai Key Laboratory of Stomatology, Shanghai 200011, China; 4 Department of Oral and Maxillofacial-Head and Neck Oncology, Ninth People’s Hospital, Shanghai Jiao Tong University School of Medicine, Shanghai 200011, China; 5 School & Hospital of Stomatology, Tongji University, Shanghai Engineering Research Center of Tooth Restoration and Regeneration, Shanghai 200072, China

## Abstract

It is a big challenge for bone healing under osteoporotic pathological condition with impaired angiogenesis, osteogenesis and remodeling. In the present study, the effect of Ca, Mg, Si containing akermanite bioceramics (Ca_2_MgSi_2_O_7_) extract on cell proliferation, osteogenic differentiation and angiogenic factor expression of BMSCs derived from ovariectomized rats (BMSCs-OVX) as well as the expression of osteoclastogenic factors was evaluated. The results showed that akermanite could enhance cell proliferation, ALP activity, expression of Runx2, BMP-2, BSP, OPN, OCN, OPG and angiogenic factors including VEGF and ANG-1. Meanwhile, akermanite could repress expression of osteoclastogenic factors including RANKL and TNF-α. Moreover, akermanite could activate ERK, P38, AKT and STAT3 signaling pathways, while crosstalk among these signaling pathways was evident. More importantly, the effect of akermanite extract on RANKL-induced osteoclastogenesis was evaluated by TRAP staining and real-time PCR assay. The results showed that akermanite could suppress osteoclast formation and expression of TRAP, cathepsin K and NFATc1. The *in vivo* experiments revealed that akermanite bioceramics dramatically stimulated osteogenesis and angiogenesis in an OVX rat critical-sized calvarial defect model. All these results suggest that akermanite bioceramics with the effects of Mg and Si ions on osteogenesis, angiogenesis and osteoclastogenesis are promising biomaterials for osteoporotic bone regeneration.

Osteoporosis has become one of the most universal and complex skeletal disorders for postmenopausal women, the elderly and those associated with other medical conditions or as the result of certain therapeutic interventions, which now affects over 200 million people worldwide^1^,^2^. Osteoporosis is characterized by low bone mass, poor bone strength and microarchitectural deterioration of bone, which is attributed to an excessive osteoclastic bone resorption and a reduced capacity of osteoblasts to replace the resorbed bone^3^,^4^. Under osteoporotic pathological condition, the patients may face increased risks of fractures and the bone defects resulted from fracture, metastasis bone tumor resection, and arthroplasty revision of the knee and hip^5^. However, much attention in both research and clinical study is focused on fracture prevention and in the development of therapeutic approaches for the enhancement of bone density and bone mass, less attention has been directed to the study of the osteoporotic bone regeneration, especially in the presence of grafted biomaterials^6^.

Under osteoporotic pathological condition, the bone healing exhibits impaired angiogenesis at early stage, impaired osteogenesis at middle stage and impaired remodeling at late stage^7^. Therefore, an ideal biomaterial for osteoporotic bone regeneration should possess the abilities to promote osteogenesis and angiogenesis meanwhile inhibit osteoclastogenesis. Our previous studies have shown that Ca, Mg, Si containing akermanite bioceramics (Ca_2_MgSi_2_O_7_) could induce osteogenic differentiation of osteoblasts, bone marrow stromal cells (BMSCs) and adipose-derived stem cells (ASCs) *in vitro* and enhance bone regeneration *in vivo*^8^,^9^,^10^,^11^. Moreover, our recent studies also reported that akermanite bioceramics could improve NO synthesis and angiogenic gene expression of human aortic endothelial cells (HAECs) *in vitro* and enhance angiogenesis *in vivo*^12^,^13^. However, the outcome of these studies is only based on healthy subjects and consequently does not provide information for akermanite bioceramics applied in osteoporotic bone regeneration. Moreover, our recent study showed that silicate based bioceramics could inhibit the expression of osteoclastogenic factors, which facilitated osteoporotic bone regeneration^5^. It is suggested that akermanite bioceramics could repress the expression of osteoclastogenic factors of BMSCs under osteoporotic condition at early stage, which need to be confirmed. Moreover, as one of the key osteoclast differentiation factors, receptor activator of nuclear factor-kappa B ligand (RANKL) could mediate osteoclastogenesis and play an essential role in osteoclast differentiation^14^,^15^. However, whether akermanite bioceramics could inhibit RANKL-mediated osteoclastogenesis at late stage, needs to be systematically investigated *in vitro*.

The mitogen-activated protein kinases (MAPKs) including extracellular signal-regulated kinase (ERK), P38, and c-Jun N terminal kinase (JNK) pathways regulate cell proliferation, osteoblast differentiation and skeletal development^16^,^17^. Moreover, it is also reported that AKT signaling pathway plays an important role in the osteogenic differentiation of progenitor cells as well as the angiogenic factor expression^18^,^19^,^20^. Signal transducer and activator of transcription 3 (STAT3) signaling pathway plays an important role in bone development and metabolism^21^. Recent study showed that BMP-2 and dexamethasone synergistically increased alkaline phosphatase (ALP) activity via activation of STAT3 signaling in C3H10T1/2 cells^22^. More importantly, a tremendous amount of researches suggest that there is crosstalk among MAPK, AKT and STAT3 signaling pathways^23^,^24^,^25^,^26^,^27^,^28^. Previous study demonstrated that akermanite bioceramics could stimulate osteogenic differentiation of ASCs via activation of ERK signaling pathway^29^. However, whether akermanite bioceramics could activate MAPK, AKT and STAT3 signaling pathways as well as the crosstalk among these signaling pathways need to be investigated systematically.

In the preset study, our hypothesis is that the effect of akermanite bioceramic on osteogenesis, angiogenesis and osteoclastogenesis makes it to be a promising biomaterial for osteoporotic bone regeneration. In order to verify our hypothesis, the effect of akermanite extract on cell proliferation, osteogenic differentiation of BMSCs derivered from ovariectomized rats (BMSCs-OVX) as well as the expression of osteoclastogenic factors was explored by MTT, ALP activity and real-time PCR assays. Moreover, the activation of MAPK, AKT and STAT3 signaling pathways, and the crosstalk among these signaling pathways were evaluated by western blot and real-time PCR assay. Interestingly, the effect of akermanite extract on RANKL-induced osteoclastogenesis was determined by tartrate-resistant acid phosphatase (TRAP) staining and real-time PCR assay. Finally, the OVX rat critical-sized calvarial defect model was used to investigate the regulatory effect of akermanite bioceramics on the bone formation ability *in vivo*.

## Materials and Methods

### Fabrication and characterization of akermanite bioceramic scaffolds

As descried in previous studies, akermanite powders were synthesized by a sol-gel process using tetraethyl orthosilicate ((C_2_H_5_O)_4_Si, TEOS), magnesium nitrate hexahydrate (Mg(NO_3_)_2_·6H_2_O) and calcium nitrate tetrahydrate (Ca(NO_3_)_2_·4H_2_O) as raw materials, while the control β-TCP powders were synthesized by chemical precipitation method using calcium nitrate tetrahydrate (Ca(NO_3_)_2_·4H_2_O) and ammonium phosphate dibasic ((NH_4_)_2_HPO_4_)^9^,^10^,^11^. Then the β-TCP and akermanite scaffolds with diameter of 5 mm and height of 3 mm were prepared using polyethylene glycol (PEG) particulates as porogens according to our previous study^8^. The three-dimensional (3D) structures of the prepared scaffolds were observed by scanning electron microscopy (SEM, JEOL, Japan). The phase of scaffold samples was characterized by X-ray diffraction (XRD, Rigaku, Japan) with mono-chromatic CuKα radiation.

### Preparation of β-TCP and akermanite extracts

1 g of β-TCP and akermanite powders were soaked in 5 mL Dulbecco’s modified Eagle’s medium (DMEM, Gibco, USA) and incubated for 24 h, respectively. Then, the extracts were centrifuged and sterilized through a filter (Millipore, 0.22 μm). The concentrations of Ca, Mg, and Si in β-TCP and akermanite extracts were measured by inductively coupled plasma atomic emission spectroscopy (ICP-AES; Varian, USA), respectively.

### Isolation and culture of BMSCs-OVX

All animal procedures including *in vivo* animal study were performed in strict accordance with the NIH guidelines for the care and use of laboratory animals (NIH Publication No. 85e23 Rev. 1985) and approved by the Animal Research Committee of Shanghai Ninth People’s Hospital affiliated to Shanghai Jiao Tong University, School of Medicine. The experimental animals received humane care. The animals were housed in an air-conditioned environment (22 ± 2 °C), with a 12-h light/dark cycle and were allowed free access to food pellets and water throughout the experiment period. 16-week-old female Sprague-Dawley (SD) rats were given an ovariectomy through two dorsal incisions as described in previous studies^5^,^30^. After three months, OVX rat model was confirmed by measurement of body weight and bone mineral density (BMD) of lumbar; and then OVX rats were sacrificed by an overdose of pentobarbital sodium. The soft tissues on bilateral femurs were removed and the femurs were harvested under aseptic conditions. Both ends of the femurs were cut off at the metaphyses and the bone marrow was flushed out with 10 mL DMEM supplement with 10% fetal bovine serum (FBS, Gibco, USA) 100 U/mL penicillin and 100 U/mL streptomycin using a 22-gauge needle. The primary BMSCs-OVX were cultured in a humidified 37 °C and 5% CO_2_ incubator for 4 days and the medium was renewed every 2 days. When the cells reached 90% confluence, they were passaged with 0.25% trypsin/EDTA. The BMSCs-OVX of passages 2–3 were used for *in vitro* studies in the absent of additional osteogenic supplements including dexamethasone, β-glycerophosphate and ascorbic acid.

### Cell proliferation assay

To determine the optimal concentration of the extracts for following studies, various concentrations of β-TCP and akermanite extracts (1/2, 1/4, 1/8, 1/16, 1/32, 1/64 and 1/128) were used, respectively. The BMSCs-OVX were seeded in 96-well plates at 5 × 10^3^ cells/well. After 24 h, the culture medium was replaced by the medium supplemented with various concentrations of β-TCP and akermanite extracts, respectively. And then, the MTT assay was performed at days 1, 4 and 7. Briefly, MTT solution (Amresco, USA) was added and incubated for 4 h. Then, the medium was replaced with dimethyl sulfoxide (DMSO, USA) and the absorbance was measured at 490 nm by ELX Ultra Microplate Reader (Bio-tek, USA). All experiments were performed in triplicate.

### ALP assay

BMSCs-OVX were seeded in 6-well plates at a density of 8 × 10^4^ cells/well and cultured in the medium containing 1/16 concentration of β-TCP and akermanite extracts, respectively. At day 10, ALP staining was performed according to the manufacturer’s instruction (Beyotime, Jiangsu, China). More importantly, ALP activity was quantitatively determined at days 4, 7 and 10 of cell culture as following: the cells of each group were collected and resuspended in RIPA Lysis Buffer (Beyotime, China). Each sample was equivalently mixed with p-nitrophenyl phosphate (pNPP, 1 mg/mL, Sigma, USA) and quantified by absorbance at 405 nm (Bio-tek, USA) according to series of p-nitrophenol (pNP) standards. Besides, total cellular protein content for each sample was determined with the Bradford method as described in our previous study^31^. Finally, ALP activity was expressed as pNP (mM) per milligram of total cellular protein. All experiments were performed in triplicate.

### Quantitative real-time PCR assay

BMSCs-OVX were seeded in 6-well plates at a density of 8 × 10^4^ cells/well and cultured in the medium supplemented with 1/16 concentration of β-TCP and akermanite extracts, respectively. At days 4 and 7 after cell culture, total RNA for each group was isolated with Trizol reagent (Life Technologies, USA) according to manufacturer’s instructions. Then, complementary DNA (cDNA) was synthetized using a PrimeScript 1^st^ Strand cDNA Synthesis kit (Takara, Japan). Quantitative real-time PCR analysis was performed with the Bio-Rad real-time PCR system (Bio-Rad, USA) on the gene expression of runt-related transcription factor 2 (Runx2), bone morphogenetic protein 2 (BMP-2), bone sialoprotein (BSP), osteopontin (OPN), osteocalcin (OCN), osteoprotegerin (OPG), RANKL, tumor necrosis factor α (TNF-α), vascular endothelial growth factor (VEGF) and angiopoietin-1 (ANG-1). Meantime, glyceraldehyde-3-phosphatedehydrogenase (GAPDH) was acted as the housekeeping gene for normalization. The primer sequences used for rat BMSCs-OVX are listed in Table 1. All experiments were performed in triplicate.

### Western blot assay

BMSCs-OVX were seeded in 6-well plates at a density of 8 × 10^4^ cells/well and cultured in the medium supplemented with 1/16 concentration of β-TCP and akermanite extracts for 30, 60 and 90 min, respectively. At each time point, the cells were collected and lysed with a protein extraction regent (Kangchen, China). Equal amount of protein samples were resolved on sodium dodecyl sulfa-teepolyacrylamide gel electrophoresis (SDS-PAGE, Beyotime, China) and subsequently electro-transferred to a polyvinylidene difluoride membrane (PVDF, Pall, USA). The membranes were incubated with primary antibodies including rabbit anti rat ERK, P38, JNK, AKT, STAT3, phosphorylated-ERK (p-ERK), phosphorylated-P38 (p-p38), phosphorylated-JNK (p-JNK), phosphorlyted-AKT (p-AKT), phosphorlyted-STAT3 (p-STAT3) (CST, USA, dilution, 1:1000), and mouse anti rat actin (Sigma, USA, dilution, 1:5000) overnight at 4 °C. Then, the membrane for each antibody was visualized with horseradish peroxidase (HRP)-conjugated goat anti-rabbit, or rabbit anti-mouse (Beyotime, China) using the ECL plus reagents (Amersham Pharmacia Biotech, USA) under an UVItec ALLIANCE 4.7 gel imaging system, respectively. Moreover, protein band intensities on the scanned films were compared to their respective control using Quantity One Image software. The bands were firstly rounded up by the volume rect tool, and then the target area intensity was calculated. The densities of ERK, P38, JNK, AKT and STAT3 were quantified as the control group for protein expression of p-ERK, p-P38, p-JNK, p-AKT and p-STAT3, respectively.

### Signaling pathways inhibition assay

BMSCs-OVX cultured in the medium supplemented with 1/16 concentration of akermanite extract were treated by ERK signaling pathway inhibitor PD98059, P38 signaling pathway inhibitor SB202190, AKT signaling pathway inhibitor LY294002 and STAT3 signaling pathway inhibitor AG490 with a final concentration of 10 μM for 90 min, respectively. The protein expression of p-ERK, p-P38, p-JNK, p-AKT and p-STAT3 for each group was detected by western blot, and further quantitatively determined by Quantity One Image software as described previously. At day 10, ALP staining for each group was performed as described previously. Moreover, real-time PCR was performed on gene expression of Runx2, BMP-2, BSP, OPN, OCN, OPG, RANKL, TNF-α, VEGF and ANG-1 as described previously at day 7. While BMSCs-OVX cultured in the medium containing akermanite extract without any inhibitors was treated as control group.

### *In vitro* osteoclastogenesis assay

*In vitro* osteoclastogenesis assay was performed to examine the effect of akermanite extract on osteoclast differentiation. Bone marrow macrophages (BMMs) were prepared as described in previous studies^32^,^33^. Briefly, the cells were extracted from the femurs and tibias of a 6-week-old C57/BL6 mouse and incubated in complete cell culture medium containing 30 ng/mL macrophage colony-stimulating factor (M-CSF). When the medium was changed, the cells were washed to deplete residual stromal cells. After reaching 90% confluence, the cells were trypsinized for 30 min to harvest BMMs. Adherent cells on dish bottoms were classified as BMMs, and then these BMMs were plated on 96-well plates at a density of 8 × 10^3^ cells/well, after being incubated for 24 h, the cells were treated with 1/16 concentration of β-TCP and akermanite extracts containing M-CSF (30 ng/mL) and RANKL (50 ng/mL), respectively; while the cells cultured without β-TCP and akermanite extracts was treated as control group.

At day 5, the osteoclasts were fixed using 4% paraformaldehyde (PFA) and stained for TRAP activity, using an acid phosphatase kit (Sigma, USA) according to the manufacturer’s protocol without counter-staining. Image photos were obtained by Nikon microscope (Nikon, USA). The total area of TRAP-positive regions and the total number of osteoclasts were quantified on five randomly selected fields of view for each sample. Moreover, total RNA was isolated and synthesized cDNA, and real-time PCR was performed on TRAP, cathepsin K and Nuclear factor of activated T cells c1 (NFATc1) as specified previously. The primer sequences used for mouse osteoclasts are listed in Table 2. All experiments were performed in triplicate.

### *In vivo* reconstruction of calvarial defects of OVX rats

Twelve OVX rats were divided randomly into two groups for calvarial defect model as described in previous study^5^. Briefly, the rats were anaesthetized by intraperitoneal injection of pentobarbital (Nembutal 3.5 mg/100 g), a 1.0- to 1.5-cm sagittal incision was made on the scalp, and then the calvarium was exposed by blunt dissection. Two bilateral critical-sized defects were created by using a 5-mm diameter trephine bur (Fine Science Tools, USA). Finally, twenty-four critical-sized calvarial defects in twelve OVX rats were randomly filled with the β-TCP and akermanite bioceramic scaffolds, respectively. Besides, a polychrome sequential fluorescent labeling for new bone formation and mineralization was performed in six OVX rats according to our previous studies^31^,^34^. Briefly, the rats were intraperitoneally injected with 25 mg/kg tetracycline (TE, Sigma, USA), 30 mg/kg alizarin red (AL, Sigma, USA), and 20 mg/kg calcein (CA, Sigma, USA), at 2, 4 and 6 weeks after implantation, respectively.

After 8 weeks of operation, the rats, which have been injected with sequential fluorescents, were perfused with Microfil (Flowtech, USA) after euthanasia to evaluate blood vessel formation. As described in previous study, a long incision was made from the front limbs down to the xyphoid process, and then, one side of the sternum was cut and the rib cage was retracted laterally. The left ventricle was penetrated with an angiocatheter after the descending aorta was clamped. After the inferior vena cava was incised, 20 mL of heparinized saline was perfused. Subsequently, 20 mL of Microfil was perfused with a rate of 2 mL/min^35^. Finally, the defects with surrounding tissue were dissected from the host bone. All the harvested specimens were fixed in a 4% paraformaldehyde solution buffered by 0.1 M phosphate solution (pH 7.2) for 5 days before further microcomputed tomography (micro-CT) analysis and histological analysis. The other six OVX rats were sacrificed and the samples were immediately cryo-conserved in liquid nitrogen (−196 °C) and then homogenized as described in previous studies^36^,^37^. Total RNA was extracted and synthesized cDNA. Finally, real-time PCR was performed on Runx2, OCN, OPG, RANKL, TRAP and CD31 as specified previously. The primer sequences are listed in Table 1.

In the present study, the samples were further examined on by a micro-CT system (μCT-80, Scanco, Switzerland) as described in previous study^34^. Briefly, the samples were scanned using the parameters with a spot size of 7 μm and maximum voltage of 36 kV. To determine the amount of newly formed bone, the best threshold for scaffold alone was selected visually, and then a determination was made of the optimum threshold for scaffold together with newly formed bone. Moreover, the ranges and means of the gray level characteristic of scaffolds with newly formed bone were determined; consequently, the visually determined threshold to separate the scaffold from newly formed bone was set. Finally, 3D images were reconstructed and bone volume fraction (BV/TV) and trabecular thickness (Tb.Th) in the bone defect area were calculated by using its auxiliary software (Scanco Medical AG).

After being examined by micro-CT, the samples were dehydrated in ascending concentrations of alcohols from 75% to 100%, and embedded in PMMA. Three longitudinal sections for each specimen were cut into about 150 μm thick using a microtome (Leica, Germany), and then grinded and polished to a final thickness of about 40 μm. Firstly, the sections were observed for fluorescent labeling using CLSM (Leica TCS, Germany), and the fluorochrome staining for new bone formation and mineralization was quantified as described in our previous studies^31^,^34^. The number of pixels labeled with yellow (TE), red (AL), and green (CA) in each image was determined as a percentage of the mineralization area, respectively. Moreover, the labeling distance between TE and AL, AL and CA was measured and represented the mineral apposition rate at weeks 2–4 and weeks 4–6 post operation, respectively. Finally, the sections were stained with Van Gieson’s picro fuchsin for histological assay. Three randomly selected sections from the serial longitudinal sections collected from each sample were analyzed. The percentages of newly formed bone area and residual scaffold area in the whole calvarial defect area were calculated at low magnification using a personal computer-based image analysis system (Image Pro 5.0, Media Cybernetic, USA). And then, the mean values of three measurements were acted as the percentages of newly formed bone area and residual scaffold area per sample, and they were used to calculate average values for each group, respectively. Moreover, blue spots from Microfil perfusion indicated new blood vessels. Then, the area of blue spots (vessel area) was also quantitatively evaluated using the same method described previously.

### Statistical analysis

All data were expressed as means ± SD. Statistical analysis was performed by ANOVA (*in vitro* study) and Student’s T-test (*in vivo* study) using SPSS v.10.1 software (SPSS Inc, USA). Values of *p* < 0.05 were considered statistically significant.

## Results

### Material characterization

**Figure 1:**
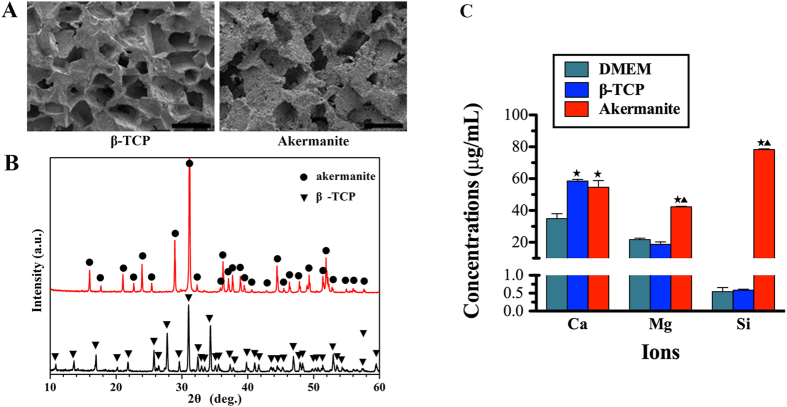
Material characterization. (**A**) SEM images of the fractured surface of the prepared β-TCP and akermanite bioceramic scaffolds; (**B**) XRD patterns of the prepared β-TCP and akermanite bioceramic scaffolds; (**C**) The concentrations of Ca, Mg and Si ions in β-TCP and akermanite extracts. ^★^indicates significant differences as compared with DMEM; ^▲^indicates significant differences as compared with β-TCP, *p* < 0.05. Scale bar = 500 μm.

The SEM images of the fractured surface of the prepared β-TCP and akermanite bioceramic scaffolds were presented in Fig. 1A. The images showed that the prepared samples were highly porous with evenly distributed and interconnected pores. The pore shape was similar to that of the PEG porogens, and the macropore sizes were about 250 ~ 400 μm. The results of the XRD patterns confirmed that the prepared scaffolds were composed of pure β-TCP and akermanite, respectively (Fig. 1B). ICP-AES analysis showed that the similar concentration of Ca was detected in β-TCP and akermanite extracts, which was higher than control DMEM medium (*p* < 0.05). More importantly, the higher concentration of Mg was detected in akermanite extract as compared with β-TCP extract and DMEM medium (*p* < 0.05), while there was no significant difference between β-TCP extract and DMEM medium. Si ion was almost no detected in β-TCP extract and DMEM medium, while the concentration of Si ion in akermanite extract was reached to about 78 μg/mL (Fig. 1C).

### Cell proliferation assay

**Figure 2:**
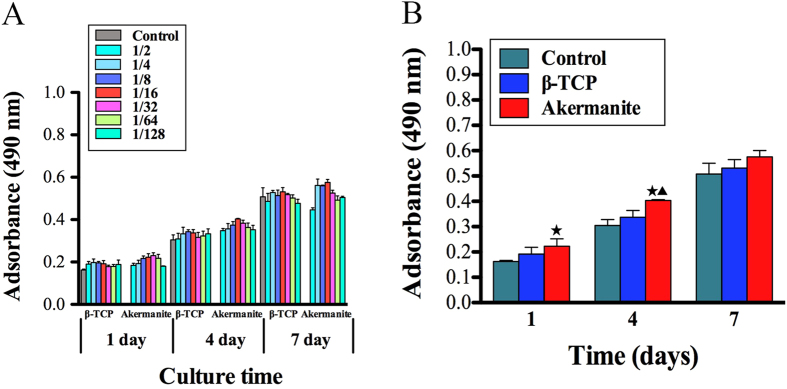
Cell proliferation assay. (**A**) MTT assay for the effect of various concentrations of β-TCP and akermanite extracts on cell proliferation of BMSCs-OVX; (**B**) The optimal concentration of 1/16 dilution of β-TCP and akermanite extracts on cell proliferation of BMSCs-OVX. The cells cultured without β-TCP and akermanite extracts was treated as control group (Control). ^★^indicates significant differences as compared with control group; ^▲^indicates significant differences as compared with β-TCP group, *p* < 0.05.

In the present study, the MTT assay was performed to compare proliferation of BMSCs-OVX cultured in various concentrations of β-TCP and akermanite extracts (Fig. 2A). It is clear to see that the cells proliferated apparently over time throughout the assay period. In addition, the proliferation of BMSCs-OVX cultured in medium supplemented with akermanite extract was greater than that for the cells cultured in either β-TCP extract or medium alone. More importantly, the results demonstrated that proliferation proceeded faster when BMSCs-OVX were cultured in a 1/16 dilution of the extracts, which had significant difference between akermanite and medium alone at days 1 and 4, akermanite and β-TCP at day 4 (*p* < 0.05) (Fig. 2B). Based on the results of MTT analysis, the 1/16 dilution of β-TCP and akermanite extracts was chosen as the appropriate concentration for the following studies.

### ALP assay

**Figure 3:**
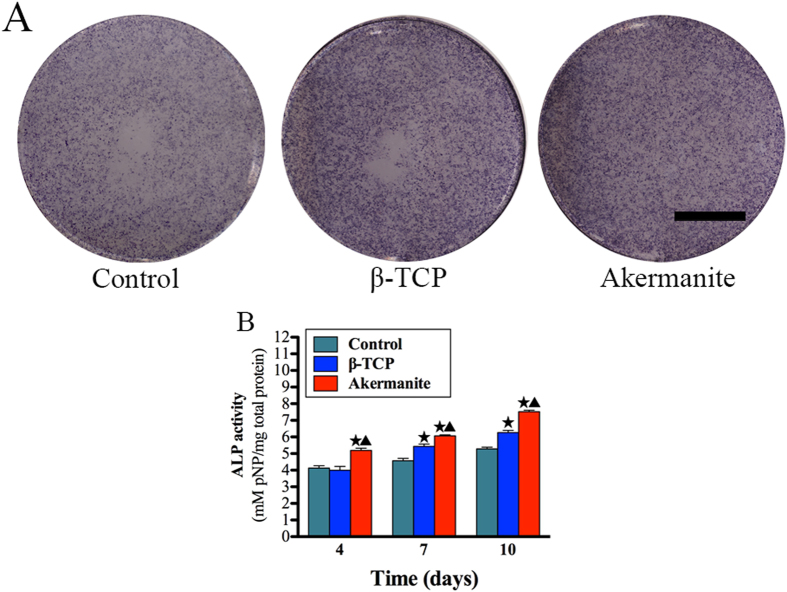
ALP activity assay. (**A**) ALP staining of BMSCs-OVX treated with β-TCP and akermanite extracts for 10 days; (**B**) ALP quantitative assay of BMSCs-OVX treated with β-TCP and akermanite extracts at days 4, 7 and 10. The cells cultured without β-TCP and akermanite extracts was treated as control group (Control). ^★^indicates significant differences as compared with control group; ^▲^indicates significant differences as compared with β-TCP group, *p* < 0.05. Scale bar = 10 mm.

As shown in Fig. 3A, ALP staining for OVX-BMSCs cultured in akermanite extract was more intensive than that for the cells cultured in β-TCP extract and medium alone at day 10. More importantly, the results of ALP quantitative analysis revealed that the ALP activity of the cells cultured in akermanite extract was significantly higher than that for the cells cultured in β-TCP extract and medium alone at days 4, 7 and 10 (*p* < 0.05). Besides, there was also significant difference between the cells cultured in β-TCP extract and medium alone at days 7 and 10 (*p* < 0.05) (Fig. 3B).

### Quantitative real-time PCR assay

**Figure 4:**
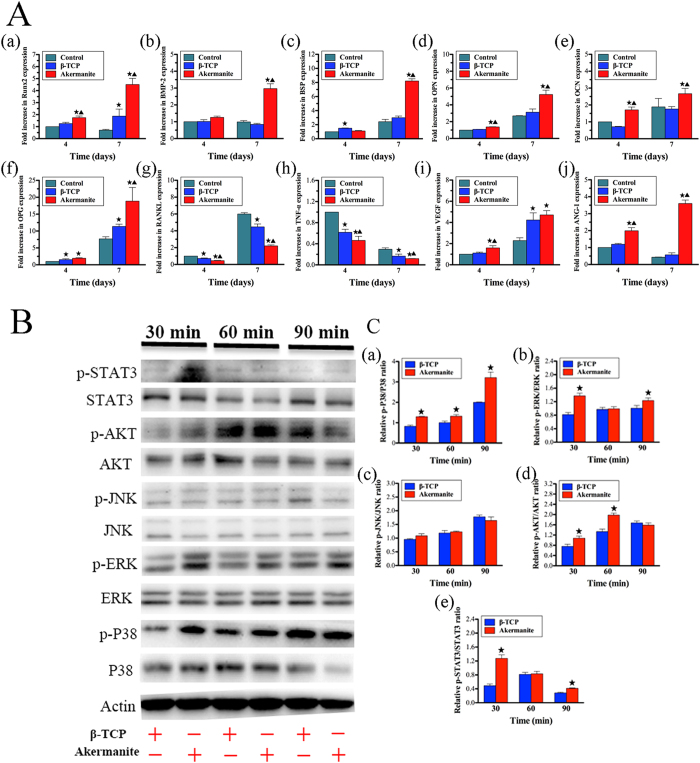
The effect of akermanite extract on the expression of osteogenic, angiogenic and osteoclastogenic genes and the activation of signaling pathways. (**A**) Rea-time PCR analysis of Runx2 (a), BMP-2 (b), BSP (c), OPN (d), OCN (e), OPG (f), RANKL (g), TNF-α (h), VEGF (i) and ANG-1 (j) in BMSCs-OVX treated with β-TCP and akermanite extracts at days 4 and 7. The cells cultured without β-TCP and akermanite extracts was treated as control group (Control). ^★^indicates significant differences as compared with control group; ^▲^indicates significant differences as compared with β-TCP group, *p* < 0.05; (**B**) Western blot assay for key protein expression of MAPK, AKT and STAT3 signaling pathways for BMSCs-OVX treated with β-TCP and akermanite extracts at 30, 60 and 90 min; (**C**) The quantitative assay for the ratios of p-P38/P38 (a), p-ERK/ERK (b), p-JNK/JNK (c), p-AKT/AKT (d) and p-STAT3/STAT3 (e). ^★^indicates significant differences as compared with β-TCP group, *p* < 0.05.

In the present study, the expression of osteogenic, osteoclastogenic and angiogenic genes for BMSCs-OVX cultured in β-TCP and akermanite extracts, was detected by quantitative real-time PCR assay at days 4 and 7. As shown in Fig. 4A, akermanite could enhance the expression of Runx2, OPN and OCN at days 4 and 7, BMP-2 and BSP at day 7 as compared with either medium alone or β-TCP (*p* < 0.05). Besides, β-TCP could also promote the expression of Runx2 at day 7, BSP at day 4 as compared with medium alone (*p* < 0.05). Moreover, both β-TCP and akermanite could enhance the expression of OPG, meantime inhibit the expression of RANKL and TNF-α at days 4 and 7, while there was also significant difference for OPG at day 4, RANKL and TNF-α at days 4 and 7 between β-TCP and akermanite (*p* < 0.05). More importantly, the higher expression of VEGF and ANG-1 was detected for BMSCs-OVX cultured in akermanite extract as compared with medium alone; there was also significant difference for VEGF at day 4, ANG-1 at days 4 and 7 between β-TCP and akermanite (*p* < 0.05). Besides, β-TCP could significantly promote the expression of VEGF at day 7 (*p* < 0.05) (Fig. 4A).

### Western blot assay

As shown in Fig. 4B, akermanite could activate P38, ERK AKT and STAT3 signaling pathways as compared with β-TCP, while akermanite have no significant effect on the activation of JNK signaling pathway. Moreover, the protein quantitative analysis showed that there was significant difference for the ratios of p-P38/P38 at 30, 60 and 90 min, p-ERK/ERK and p-STAT3/STAT3 at 30 and 90 min, p-AKT/AKT at 30 and 60 min, between the cells cultured in β-TCP and akermanite extracts (*p* < 0.05) (Fig. 4C).

### Signaling pathways inhibition assay

**Figure 5:**
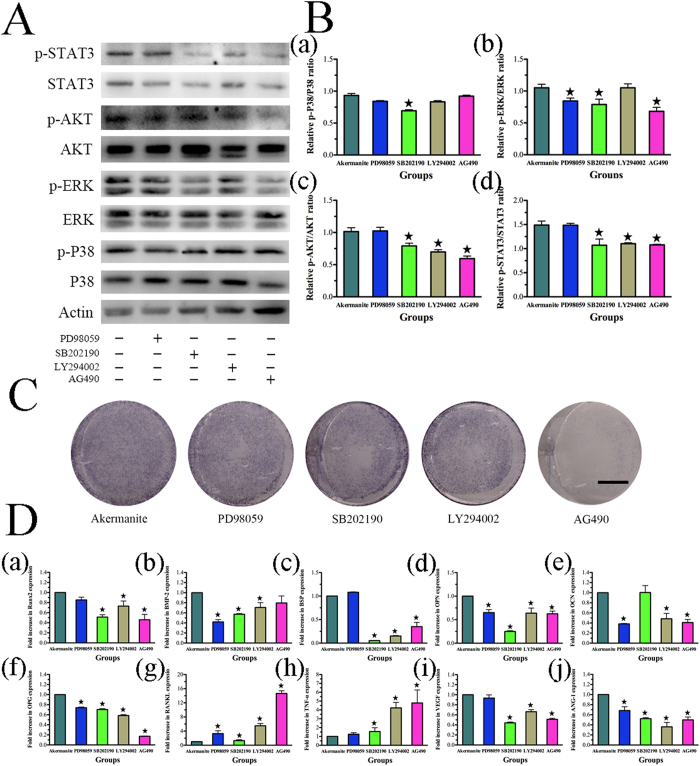
The effect of inhibitors SB202190, PD98059, LY294002 and AG490 on BMSCs-OVX treated with akermanite extract. (**A**) Western blot assay for the expression of p-P38, p-ERK, p-AKT and p-STAT3 for BMSCs-OVX cultured in akermanite extract with inhibitors SB202190, PD98059, LY294002 and AG490 for 90 min, respectively; (**B**) The quantitative assay for the ratios of p-P38/P38 (a), p-ERK/ERK (b), p-AKT/AKT (c) and p-STAT3/STAT3 (d) for BMSCs-OVX cultured in akermanite extract with inhibitors SB202190, PD98059, LY294002 and AG490 for 90 min, respectively; (**C**) ALP staining for BMSCs-OVX cultured in akermanite extract with inhibitors SB202190, PD98059, LY294002 and AG490 for 10 days, respectively; (**D**) Rea-time PCR analysis of Runx2 (a), BMP-2 (b), BSP (c), OPN (d), OCN (e), OPG (f), RANKL (g), TNF-α (h), VEGF (i) and ANG-1 (j) for BMSCs-OVX cultured in akermanite extract with inhibitors SB202190, PD98059, LY294002 and AG490 for 7 days, respectively. BMSCs-OVX cultured in akermanite extract without any inhibitors was treated as akermanite group. ^★^indicates significant differences as compared with akermanite group, *p* < 0.05. Scale bar = 10 mm.

The results of western blot showed that the phosphorylation of P38, ERK, AKT and STAT3 for BMSCs-OVX treated with akermanite extract could be repressed by inhibitors SB202190, PD98059, LY29402 and AG490, correspondingly (Fig. 5A,B). Moreover, the P38 inhibitor SB202190 could inhibit the phosphorylation of ERK, AKT and STAT3, while STAT3 inhibitor AG490 could inhibit the phosphorylation of ERK and AKT. Besides, the phosphorylation of STAT3 could also be repressed by the AKT inhibitor LY294002 (*p* < 0.05) (Fig. 5A,B). Besides, ALP activity and the gene expression of Runx2, BMP-2, BSP, OPN, OCN, OPG, VEGF and ANG-1 could be repressed by inhibitors SB202190, PD98059, LY294002 and AG490, respectively, while the gene expression of RANKL and TNF-α could be enhanced by these inhibitors (*p* < 0.05) (Fig. 5C,D).

### *In vitro* osteoclastogenesis assay

**Figure 6:**
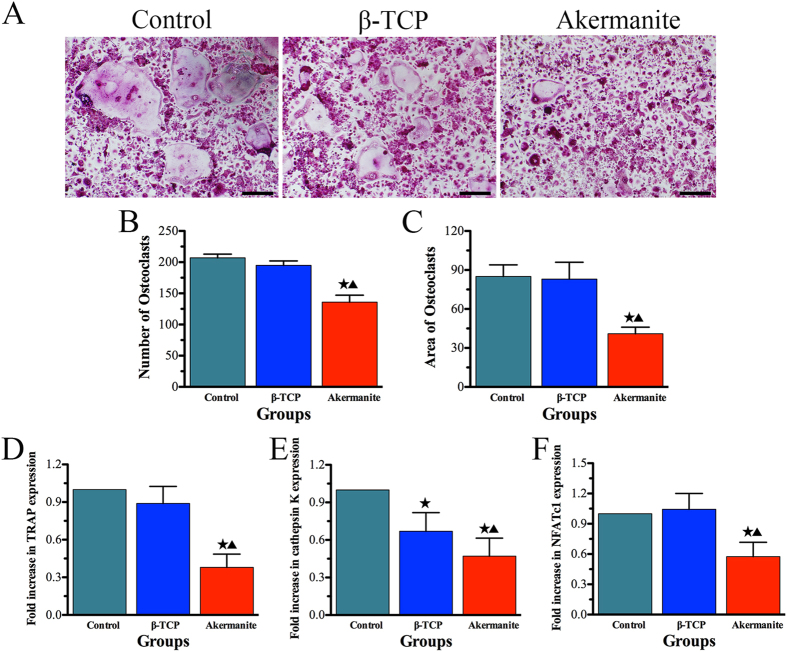
*In vitro* osteoclastogenesis assay. (**A**) TRAP staining for BMMs cultured in M-CSF and RANKL supplement with β-TCP and akermanite extracts, respectively; (**B,C**) The quantitative assay for number (**B)** and area (**C**) of osteoclasts according to TRAP staining; (**D–F**) Rea-time PCR analysis of TRAP (**D**), cathepsin K (**E)** and NFATc1 (**F**) for BMMs cultured in M-CSF and RANKL supplement with β-TCP and akermanite extracts, respectively. The cells cultured in M-CSF and RANKL without β-TCP and akermanite extracts was treated as control group (Control). ^★^indicates significant differences as compared with control group; ^▲^indicates significant differences as compared with β-TCP group, *p* < 0.05. Scale bar = 100 μm.

In the present study, the effect of akermanite on osteoclast formation was also systematically evaluated *in vitro*. As shown in Fig. 6A–C, numerous TRAP-positive multinucleated osteoclasts formed in the control and β-TCP groups. However, the number of mature osteoclasts was decreased significantly for the cells cultured with akermanite extract (*p* < 0.05). Moreover, quantitative real-time PCR assay was applied to analyze and quantify the RANKL-induced mRNA expression of osteoclast-related genes (TRAP, cathepsin K and NFATc1) in order to confirm the inhibitory effect of akermanite on osteoclast formation. The results showed that the gene expression of TRAP, cathepsin K and NFATc1 could be suppressed by akermanite as compared with either control group or β-TCP group (*p* < 0.05). Besides, β-TCP could also inhibit the gene expression of cathepsin K as compared with control group (*p* < 0.05) (Fig. 6D–F).

### *In vivo* regeneration of calvarial defects of OVX rats

**Figure 7:**
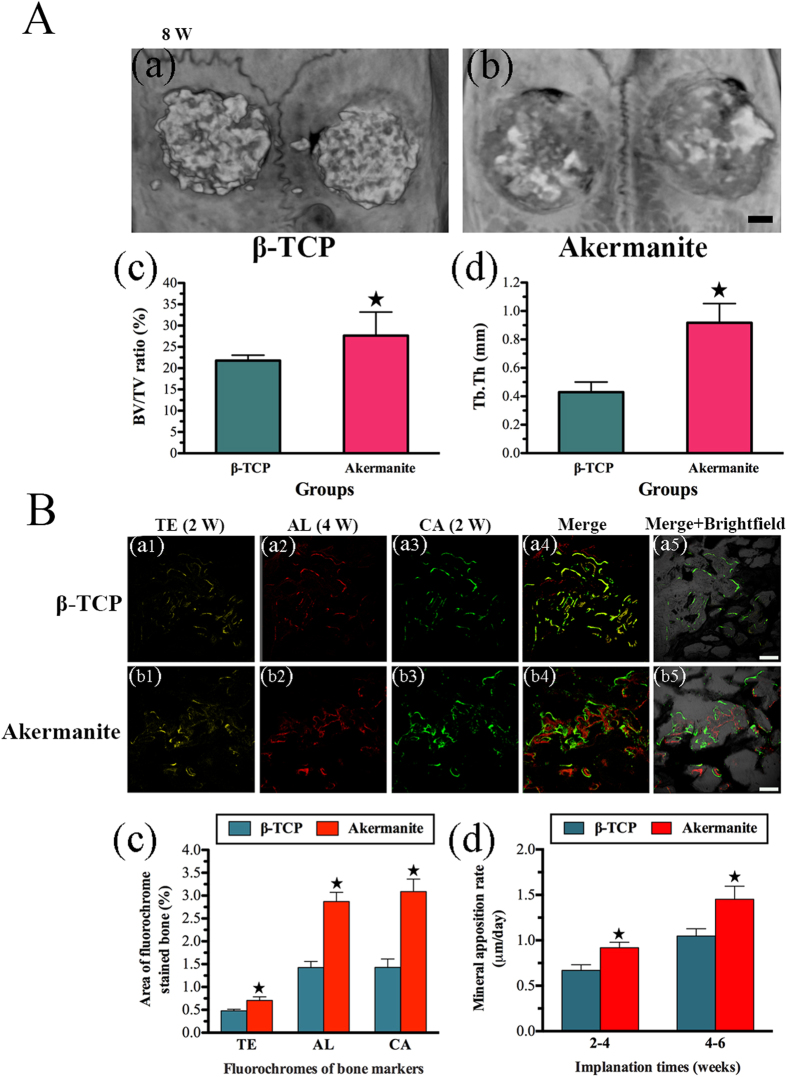
Micro-CT assay and sequential fluorescent labeling assay for *in vivo* bone regeneration. (**A**) Representative micro-CT 3D superficial images of new bone formation (a: β-TCP; b: akermanite) and morphometric analysis of BV/TV (c) and Tb.Th (d) for each group at 8 weeks postoperation; (**B**) The images in yellow (TE; a1, b1), red (AL; a2, b2) and green (CA; a3, b3) represented bone formation and mineralization at 2, 4 and 6 weeks after operation, while the images of a4–a5 and b4–b5 represented the merged images of the three fluorochromes, or together with a brightfield confocal laser microscope image for β-TCP (a1–a5) and akermanite (b1–b5) groups, respectively; The percentages of TE, AL and CA staining (c) and mineral apposition rate (d) of 2–4 weeks and 4–6 weeks for β-TCP and akermanite groups assessed at week 8 after implantation by histomorphometric analysis. ^▲^indicates significant differences between β-TCP and akermanite groups, *p* < 0.05. A: Scale bar = 1 mm; B: Scale bar = 100 μm.

As shown in Fig. 7A, the newly formed bone reconstructed by micro-CT in akermanite group was greater than that in β-TCP group. Moreover, the morphometrical analysis showed that significantly greater BV/TV and Tb.Th were detected in akermanite group (30.15 ± 4.33% and 0.92 ± 0.14 mm, respectively) as compared with β-TCP group (21.78 ± 1.28% and 0.43 ± 0.071 mm, respectively) (*p* < 0.05) (Fig. 7A).

In the present study, polychrome sequential fluorescent labeling was conducted to evaluate the stages of bone formation at weeks 2, 4 and 6 (Fig. 7B). At week 2, the percentage of TE labeling (yellow) in akermanite group (0.71 ± 0.19%) was significantly higher than that in β-TCP group (0.48 ± 0.08%) (*p* < 0.05). At week 4, the percentage of AL labeling (red) for akermanite group reached 2.87 ± 0.49%, which was higher than that in β-TCP group (1.43 ± 0.32%) (*p* < 0.05). The higher percentage of CA labeling (green) was detected in akermanite group (3.09 ± 0.67%) as compared with β-TCP group (1.43 ± 0.45%) at week 6 (*p* < 0.05). More importantly, at weeks 2–4 post operation, the mineral apposition rate (labeling distance between TE and AL) for akermanite group (0.92 ± 0.16 μm/day) was higher than that in β-TCP group (0.67 ± 0.15 μm/day) (*p* < 0.05). At weeks 4–6 post operation, the higher mineral apposition rate (labeling distance between AL and CA) was detected in akermanite group (1.45 ± 0.35 μm/day) as compared with β-TCP group (1.05 ± 0.19 μm/day) (*p* < 0.05) (Fig. 7B).

**Figure 8:**
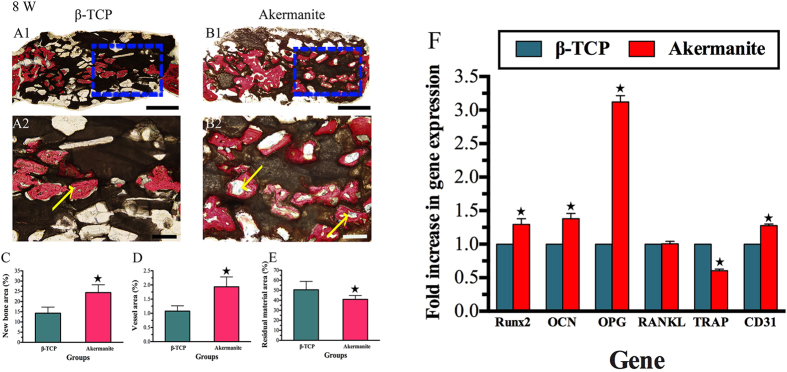
Histological and histomorphometric assay. (**A,B**) Histological images of newly formed bone in β-TCP (A1–A2) and akermanite (B1–B2) groups at week 8 after operation; (**C–E**) The percentage of new bone area (**C**), vessel area (**D**) and residual material area (**E**) assessed for β-TCP and akermanite groups by histomorphometric analysis; (**F**) Real-time PCR for the expression of Runx2, OCN, OPG, RANKL, TRAP and CD31 in calvarial defect area. ^★^indicates significant differences between β-TCP and akermanite groups, *p* < 0.05. A1, B1: Scale bar = 1 mm; A2, B2: Scale bar = 100 μm.

The histological images showed that that much more newly formed bone was achieved in akermanite group as compared with β-TCP group (Fig. 8A1-B1). More importantly, the histomorphometric assay further demonstrated that the percentage of new bone area in akermanite group (24.38 ± 3.80%) was much higher than that in β-TCP group (14.29 ± 2.98%) (*p* < 0.05) (Fig. 8C). Moreover, as shown in Fig. 8A2-B2, each blue spot (yellow arrow) from Microfil perfusion represent a new formed blood vessel, which suggested that more newly formed blood vessels were observed in akermanite group as compared with β-TCP group. The histomorphometric assay confirmed that the higher percentage of new vessel area was observed in akermanite group (1.93 ± 0.35%) as compared with β-TCP group (1.08 ± 0.19%) (*p* < 0.05) (Fig. 8D). Besides, it could be seen that 40.92 ± 3.90% of residual akermanite remained, while 50.48 ± 8.43% of residual β-TCP still remained after 8 weeks of implantation *in vivo* (*p* < 0.05) (Fig. 8E). More importantly, real-time PCR showed that the enhanced expression of Runx2, OCN, OPG and CD31 was detected in calvarial defect area of akermanite group as compared with β-TCP group. The expression of TRAP was drastically decreased in akermanite group as compared with β-TCP group; however, there was no significant difference on the expression of RANKL (*p* < 0.05) (Fig. 8F).

## Discussion

Osteoporosis is a metabolic condition characterized by an inadequate bone formation and excessive bone resorption, which could lead to decreased bone mass and microarchitectural deterioration of the skeleton^6^. Moreover, the bone healing of drill-hole defect in OVX mice model exhibited impaired angiogenesis at early stage, impaired osteogenesis at middle stage and impaired remodeling at late stage, which could result in compromised mechanical property in the end^7^. Therefore, it is urgent to develop novel therapeutical strategies for osteoporotic bone healing. Recent studies have focused on designing the chemical composition of grafted biomaterials to facilitate bone repair^38^,^39^. However, the outcome of these studies is only based on healthy subjects, and does not provide information on bone substitute material performance in osteoporotic situation^40^. It is suggested that a novel biomaterial with optimal chemical composition could meet the demands of bone regeneration in osteoporotic situation such as enhanced abilities to promote osteogenesis/angiogenesis meanwhile suppress osteoclastogenesis.

Our previous studies have designed and fabricated Ca, Mg, Si containing akermanite bioceramics (Ca_2_MgSi_2_O_7_), which showed controllable degradation rate and desirable mechanical properties^41^,^42^. Moreover, it has been reported that akermanite bioceramics could induce osteogenic differentiation of osteoblasts, BMSCs and ASCs *in vitro* and enhanced bone regeneration *in vivo* under healthy condition^8^,^9^,^10^,^11^. Our recent study also showed that akermanite bioceramics possessed distinctive dual functions of osteogenesis/angiogenesis stimulation *in vitro* and *in vivo*^12^. Moreover, our recent study reported that Si based bioceramics could repress the expression of osteoclastogenic factors *in vitro* and enhance osteoporotic bone regeneration *in vivo*^5^. However, whether Ca, Mg, Si containing akermanite bioceramics could have inhibitory effect on osteoclastogenesis, consequently could act as promising grafted biomaterials for osteoporotic bone repair, was largely unknown.

In the present study, BMSCs, which derived from osteoporotic rats, were cultured and treated with β-TCP and akermanite extracts. Previous studies showed that the decreased proliferation ability and osteogenic differentiation potential was detected in OVX BMSCs as compared with sham BMSCs^43^,^44^. Meantime, estrogen deficiency in postmenopausal osteoporosis condition could increase the production of RANKL and inhibit OPG secretion of stromal cells/preosteoblasts^45^. Consequently, the reduced osteoblastic differentiation and enhanced osteoclastic differentiation abilities of BMSCs derived from osteoporotic patients, which ultimately caused the delay of bone formation or nonunion^46^,^47^. Present study showed that akermanite could enhance ALP activity and the gene expression of osteogenic makers (Runx2, BMP-2, BSP, OPN and OCN). More importantly, RANKL, as a critical gene in osteoclastogenesis, could induce osteoclast differentiation, activation, and survival upon interaction with its receptor RANK; meanwhile, OPG is a competitive inhibitor of RANKL and thus blocks RANKL from activating osteoclasts^48^. The ovariectomy-associate bone loss in osteoporosis could be attributed to the misbalance of RANKL/OPG system^49^. Our results showed that akermanite bioceramics could inhibit the expression of RANKL meanwhile enhance the expression of OPG as comparing with either β-TCP or control group, which means that akermanite could rebuilt the balance of RANKL/OPG system and rescue of impaired bone healing in osteoporotic situation. Besides, TNF-α, which is pivotal to the pathogenesis of inflammatory osteolysis, could directly promote osteoclastic differentiation and activation, and synergize with RANKL for both^50^,^51^. Present study showed that both β-TCP and akermanite bioceramics could repress the expression of TNF-α as compared with control group, while akermanite had the stronger inhibitory effect.

In the present study, we also evaluated the effect of akermanite bioceramics on RANKL-induced osteoclastogenesis. It is well known that cathepsin K and TRAP are related to the bone resorptive function of mature osteoclasts^52^,^53^; NFATc1 known as a master regulator of osteoclast differentiation, can regulate the expression of a number of osteoclast specific genes, such as TRAP and cathepsin K^54^. Interestingly, the results from TRAP staining and RT-PCR on gene expression of TRAP, cathepsin K and NFATc1 showed that β-TCP bioceramics might have slightly inhibited effect on osteoclast formation, while akermanite bioceramics could significantly suppress osteoclast formation. Based on these findings, it is suggested that akermainite bioceramics may not only have inhibited effect on the expression of RANKL and TNF-α of OVX-BMSCs at early stage, but also could repress RANKL-induced osteoclastogenesis at late stage. Moreover, it is known that the RANKL-RANK-activated NF-κB signaling pathway induces NFATc1 gene expression, and Ca^2+^-dependent calcineurin signaling plays a critical role in NFATc1 auto-amplification^55^,^56^. Previous study showed that Mg ion could inhibit RANKL-induced osteoclastogenesis via blocking the NF-κB signaling pathway and preventing Ca^2+^-dependent calcineurin signaling pathway^33^. However, whether the inhibited effect of akermanite on RANKL-induced osteoclastogenesis is related to NF-κB and Ca^2+^-dependent calcineurin signaling pathways, need to be further evaluated in our future study.

Angiogenesis is the basic step in the process of bone regeneration, which provides blood supply and consequently benefits the subsequent progress of osteogenesis^57^. It has been shown that angiogenesis occurred before osteogenesis in the healing of bone defects, and then both angiogenesis and osteogenesis anticipated in bone regeneration and promote the effect of each other^58^. Moreover, angiogenic factors play an important role in these two processes^17^. As a key angiogenic factor, VEGF could induce mobilization and recruitment, proliferation and differentiation of endothelial progenitor cells (EPC), as well as the recruitment and survival of osteoblasts^59^,^60^. Meanwhile, ANG-1 acting as another important angiogenic factor could stimulate angiogenesis by reducing the VEGF-mediated vascular permeability to a certain extent^61^. Moreover, previous studies reported that VEGF expression is notably reduced in multiple cell types, including mesenchymal stem cells, with age; moreover; reduced VEGF expression in mesenchymal stem cells resulted in reduced osteoblast differentiation^62^,^63^. Present study showed that akermanite bioceramics could enhance the expression of VEGF and ANG-1 of OVX-BMSCs as compared with both β-TCP and control groups, which may rescue of impaired angiogenetic capacity and facilitate bone healing in osteoporotic situation. Based on these results, it is suggested that akermanite bioceramics could induce osteogenic differentiation of OVX-BMSCs as well as the angiogenic factor expression. Moreover, akermanite not only inhibited the expression of RANKL and TNF-α of OVX-BMSCs at early stage, but also repressed RANKL-induced osteoclastogenesis at late stage. Most studies have shown that both Mg and Si ions could enhance cell proliferation and osteogenic differentiation alone^64^,^65^. Moreover, recent studies reported that either Si or Mg ions could inhibit osteoclast formation *in vitro*^33^,^66^. More importantly, it is demonstrated in recent studies that Mg ions could stimulate the production of VEGF of human BMSCs, while Si ions could up-regulated the expression of VEGF of human umbilical vein endothelial cells (HUVECs)^67^,^68^. In the present study, the similar concentration of Ca was detected in β-TCP and akermanite extracts, while higher concentrations of Mg and Si ions were detected in akermanite extract as compared with β-TCP extract. It is suggested that the enhanced effect of Mg and Si ions on osteogenesis and angiogenesis, as well as inhibited effect on osteoclastogenesis make akermanite to be an excellent candidate as grafted biomaterial for osteoporotic bone regeneration.

**Figure 9:**
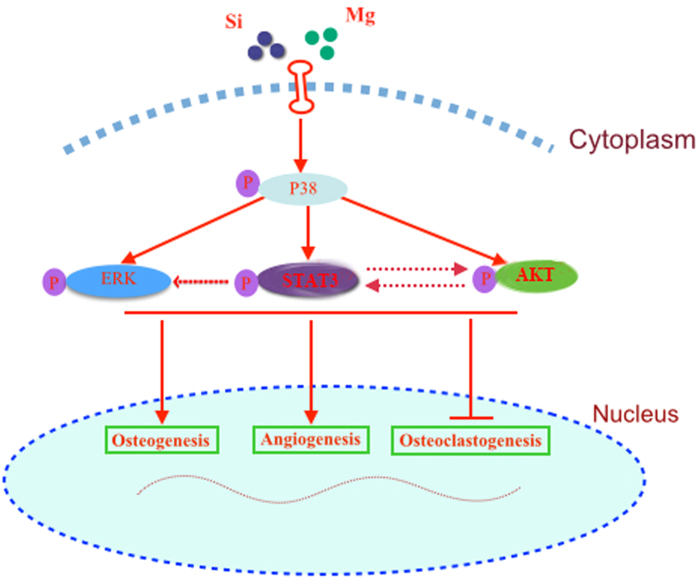
The crosstalk among P38, ERK, AKT and STAT3 signaling pathways for the effect of akermanite bioceramics on BMSCs-OVX.

It is well known that MAPK signaling pathways including ERK, P38 and JNK signaling pathways are crucial for the regulation of cell proliferation, osteoblast differentiation and skeletal development^69^,^70^. Moreover, AKT signaling pathway, which plays a key role in the physiology and pathophysiology of various types of cells, could exert profound effect on diverse processes including cell proliferation, migration, metabolism and differentiation^71^,^72^. Recent studies reported that AKT signaling pathway played an important role in the osteogenic differentiation of progenitor cells as well as the angiogenic factor expression^18^,^19^,^20^. STAT3 signaling pathway plays an important role in bone development and metabolism^21^. Previous study showed that STAT3 mutation caused a disease called Job syndrome, and most patients with that disease have associated craniofacial and skeletal features^73^. More importantly, it is reported that BMP-2 and dexamethasone synergistically increased ALP level via activation of STAT3 signaling in C3H10T1/2 cells^22^. Present study showed that akermanite bioceramics could activate P38, ERK, AKT and STAT3 signaling pathways as compared with β-TCP bioceramics. While the effect of akermanite on OVX-BMSCs could be repressed by these signaling pathway inhibitors, respectively. Besides, luciferase assay in previous study using ALP promoter deletion constructs showed that a region of the promoter containing a putative STAT3 response element (SRE) responds to C3H10T1/2 cells treatment with a combination of BMP-2 and Dex. Moreover, ChIP assay indicated that STAT3 bound to the SRE and STAT3 siRNA suppressed the synergistic effect of BMP-2 and Dex on ALP level^22^. It is suggested that STAT3 may play an important role in regulating ALP expression. Present study also showed that ALP activity of OVX-BMSCs cultured in akermanite extract could be dramatically repressed by AG490 treatment as compared with the other signaling pathway inhibitor treatment. However, previous study reported that akermanite bioceramics stimulated osteogenic differentiation of ASCs via activation of ERK pathway while there was no detectable activation of P38 signaling pathway^29^. This phenomenon may be attributed to the different cell type tested in the present study. More importantly, present study also showed that P38 signaling inhibitor could also reduce phosphorylation of ERK, AKT and STAT3, which suggested that P38 signaling pathway might be upstream of ERK, AKT and STAT3 signaling pathways^23^,^24^,^25^,^74^,^75^. Previous studies have reported that there was also crosstalk among ERK, AKT and STAT3 signaling pathways^26^,^28^. Present study also showed that the STAT3 inhibitor could inhibit the phosphorylation of ERK and AKT, while the AKT inhibitor could repress the phosphorylation of STAT3. It is suggested that there is crosstalk among ERK, AKT and STAT3 signaling pathways as shown in Fig. 9. Moreover, it is known that Src homology and collagen (Shc) is a common point of integration between bone morphogenetic protein 2, AKT pathway and the Ras/MAPK pathway^76^,^77^,^78^. Moreover, recent studies reported that β-TCP-Mg-3AI-1Zn surface could enhance activation of Shc, and consequently activated Ras/Raf-MAPK cascade^77^. Whether Mg and Si containing akermanite could activate Shc, and consequently affect the phosphorylation of STAT3, AKT, ERK and P38, need to be evaluated in future.

Previous study has reported that akermanite bioceramics could promote osteogenesis and angiogenesis as compared with β-TCP bioceramics in rabbit femur defect models^13^. However, recent study has shown that both impaired bone formation and disruption of bone remodeling equilibrium in osteoporosis condition seemed to converge to limit the bone regeneration in OVX rat subcritical-size calvarial defects^79^. Therefore, the results from studies based on healthy subjects may not provide information for akermanite bioceramics applied in osteoporotic bone regeneration. In the present study, OVX rat critical-size calvarial defect models were applied to evaluate the bone repair effect of akermanite bioceramics. The higher value of BV/TV, Tb.Th and new bone area was detected in akermanite group as compared with β-TCP group. Moreover, widespread distribution of vascularization was also detected throughout akermanite bioceramics, which possessed higher newly formed blood vessel area as compared with β-TCP bioceramics. More importantly, real-time PCR for calvarial defect area showed that akermanite bioceramics could enhance the expression of osteogenic markers (Runx2 and OCN); however, there was no significant difference on the expression of RANKL, akermanite could also drastically enhance the expression of OPG meantime inhibit the expression of TRAP. It is also confirmed that akermanite bioceramics could rebuilt the balance of RANKL/OPG system and inhibit osteoclastogenesis *in vivo*. CD31 also known as platelet endothelial cell adhesion molecule-1 (PECAM-1), makes up a large portion of endothelial cell intercellular junctions and plays an important role in angiogenesis; moreover, it is also acted as an endothelial cell marker^80^,^81^. In the present study, akermanite bioceramics could significantly enhance the expression of CD31 as compared with β-TCP bioceramics, which verified that akermanite bioceramics could promote angiogenesis *in vivo*. All these *in vivo* results suggested that akermanite bioceramics could be acted as grafted materials for osteoporotic bone regeneration due to the stimulation of both bioactive Mg and Si ions. However, a large animal model with the longer implantation periods is needed in future to evaluate the bone repair effect of akermanite bioceramics under osteoporotic condition.

## Conclusions

Present study demonstrated that akermanite bioceramics enhanced cell proliferation and osteogenic differentiation of OVX-BMSCs as well as the expression of angiogenic factors. Moreover, akermanite bioceramics inhibited the expression of RANKL and TNF-α of OVX-BMSCs at early stage, and repress RANKL-induced osteoclastogenesis at late stage. The effect of akermanite on OVX-BMSCs might be related to P38, ERK, AKT and STAT3 signaling pathways, while crosstalk among these signaling pathways was also evident. *In vivo* study using critical-size calvarial defects of OVX rat models showed that akermanite bioceramics not only possessed superior osteoinductive activity to enhance bone formation, but also stimulated angiogenesis and inhibited osteoclastogenesis as compared with β-TCP bioceramics. It is suggested that the effect of Mg and Si ions on osteogenesis, angiogenesis and osteoclastogenesis make akermanite bioceramics to be promising biomaterials for osteoporotic bone regeneration.

## Additional Information

**How to cite this article**: Xia, L. *et al.* Akermanite bioceramics promote osteogenesis, angiogenesis and suppress osteoclastogenesis for osteoporotic bone regeneration. *Sci. Rep.* **6**, 22005; doi: 10.1038/srep22005 (2016).

## Figures and Tables

**Table 1:** Primer sequences used for rat BMSCs-OVX.

Gene	Primers (F = forward; R = reverse)	Accession numbers	Product size (bp)
Runx2	F: 5′ATCCAGCCACCTTCACTTACACC3′	NM_053470.2	199
	R: 5′GGGACCATTGGGAACTGATAGG3′		
BMP-2	F: 5′GAAGCCAGGTGTCTCCAAGAG3′	NM_017178.1	122
	R: 5′GTGGATGTCCTTTACCGTCGT3′		
BSP	F: 5′AGAAAGAGCAGCACGGTTGAGT3′	NM_012587.2	175
	R: 5′ GACCCTCGTAGCCTTCATAGCC3′		
OPN	F: 5′CCAAGCGTGGAAACACACAGCC3′	NM_012881.2	165
	R: 5′GGCTTTGGAACTCGCCTGACTG3′		
OCN	F: 5′CAGTAAGGTGGTGAATAGACTCCG3′	NM_013414.1	172
	R: 5′GGTGCCATAGATGCGCTTG3′		
OPG	F: 5′GTCCCTTGCCCTGACTACTCT3′	NM_012870.2	250
	R: 5′GACATCTTTTGCAAACCGTGT3′		
RANKL	F: 5′CCCATCGGGTTCCCATAAAGTC3′	NM_057149.1	146
	R: 5′GCCTGAAGCAAATGTTGGCGTA3′		
TNF-α	F: 5′GCGTGTTCATCCGTTCTCTA3′	NM_012675.3	198
	R: 5′ACTACTTCAGCGTCTCGTGTGT3′		
TRAP	F: 5′GTGCATGACGCCAATGACAAG3′	XM_006242694.2	98
	R: 5′TTTCCAGCCAGCACGTACCA3′		
VEGF	F: 5′GGCTCTGAAACCATGAACTTTCT3′	NM_001110334.1	165
	R: 5′GCAGTAGCTGCGCTGGTAGAC3′		
ANG-1	F: 5′GGACAGCAGGCAAACAGAGCAGC3′	NM_053546.1	130
	R: 5′CCACAGGCATCAAACCACCAACC3′		
CD31	F: 5′GCTGTCTACTCAGTCATGGCC3′	NM_031591.1	231
	R: 5′CGTCTCTTCCTTCTGGATGGTG3′		
GAPDH	F: 5′CCTGCACCACCAACTGCTTA3′	NM_017008.4	120
	R: 5′GGCCATCCACAGTCTTCTGAG3′		

**Table 2:** Primer sequences used for mouse osteoclasts.

Gene	Primers (F = forward; R = reverse)	Accession numbers	Product size (bp)
TRAP	F: 5′CTGGAGTGCACGATGCCAGCGACA3′	NM_001102405.1	419
	R: 5′TCCGTGCTCGGCGATGGACCAGA3′		
cathepsin K	F: 5′CTTCCAATACGTGCAGCAGA3′	NM_007802.4	155
	R: 5′TCTTCAGGGCTTTCTCGTTC3′		
NFATc1	F: 5′CCGTTGCTTCCAGAAAATAACA3′	NM_016791.4	152
	R: 5′TGTGGGATGTGAACTCGGAA3′		
β-actin	F: 5′TCTGCTGGAAGGTGGA3′	NM_007393.4	188
	R: 5′CCTCTATGCCAACACAGTGC3′		
